# The Loyalty of Society Members

**Published:** 1895-03

**Authors:** 


### Editorial.

### THE LOYALTY OF SOCIETY MEMBERS.

It may be held that the principal purpose of dental association is educational. While there are other influences which are beneficial, particularly the attendant social intercourse, it must be conceded that the advancement in the study of scientific principles, the acquisition and relation of facts, and the understanding of practical procedures are the chief ends in view.

Each society in a certain sense is representative of a human mind. If it pursues its work with the definite purpose of carrying out these fundamental objects, it acquires an individuality and character of its own. Where all are animated by the same spirit and determination to work for the benefit of the society, the greatest development in its growth will take place which its members are capable of giving to it.

This statement involves the principle that each member, and particularly those best endowed by natural capacity and acquired ability, should give to their local society their entire work in any given direction, otherwise the society is being deprived of strength and sources of growth.

Should those capable of investigation and those of clearest intellects carry their mental work to distant organizations the result is the weakening of their own society, as they are clearly depriving their associates of the benefit of the analysis and discussion of the matter.

The discouragement of having original matter with its inspiring influences coming back as secondary from some other source through publication must be depressing in its effects upon the life of any body of men.

There has grown up a practice in this country somewhat peculiar to dental societies, to induce the papers brought before them to come from leading men of other societies. Two inevitable results must be the consequence of this course,—the society from which the essayist comes is deprived of the influence upon it of the original matter to which it is entitled to have the precedence, and the society before which it is given, while it may be stimulated by a representative person in their midst and benefited by the matter, has taken a dangerous course.

This produces a species of intoxication which in its reaction weakens those who indulge in it. This is exemplified in other than dental societies. It has been stated that clubs which for intellectual purposes employ lecturers to give entertainment and instruction fail to have the same mental vigor as those which depend upon the talent within their own body. It has also been remarked, concerning some of the dental societies which have depended upon outside papers, that their members do little efficient work. To reiterate, this practice robs in both directions,—it weakens the body from which the teacher hails, and it ultimately reduces the life of the society which receives. It revives the old homely maxim, "Every tub should stand on its own bottom."

It may not be difficult to make it appear that the dispersion of efforts complained of effects the general status of the dental profession.

The dental societies of this country are arranged in three groups, the local, the State, and the national. It is evident these should work in harmony and interdependence with each other, the result then would be that scientific and practical advancement would have at last its most efficient expression in the proceedings of the national association. The representative gatherings of the profession give higher tone and should furnish stronger claims upon the public interest than the local societies. To give these satisfactory and continuous interest all advanced work should there have final consideration. The discussion would then have a wider range and be subject to the determination of the more experienced members of the profession. At present new methods and new remedies are exploited and widely put in practice, upon the authority of individuals, without the previous discussion necessary to put them into digestible form; witness, for examples, the pernicious wave of copper amalgam, and that remedy after remedy is brought forward for given purposes, each laid aside by the mass for the next, before its properties and mode of application to dental surgery have been clearly made out. It is our belief that this is another of the evils of diffusing efforts under conditions which restrict the winnowing and discussion which every paper and subject should have at the place and time when its real value could be made clear.

As it is to-day, the national association is woefully deficient of papers upon new subjects, and until those who represent the local and State societies bring up to it their work for final discussion it will continue to be lacking of vital interest. It should be a paramount object to sustain the character of the national association, because its proceedings qualify the world's opinions of the status of dental science and practice in this country, and should furnish a criterion for the guidance of all. We therefore hold it a necessity that each society should carefully consider all subjects brought before it, and send up its original matter to the national association to which it is related. This, as before indicated, involves the obligation that the members of each society be so loyal to its welfare as to give to it their whole mind and support. L. J.

